# Biospecimen Repositories and Integrated Databases as Critical Infrastructure for Pathogen Discovery and Pathobiology Research

**DOI:** 10.1371/journal.pntd.0005133

**Published:** 2017-01-26

**Authors:** Jonathan L. Dunnum, Richard Yanagihara, Karl M. Johnson, Blas Armien, Nyamsuren Batsaikhan, Laura Morgan, Joseph A. Cook

**Affiliations:** 1 Museum of Southwestern Biology and Biology Department, University of New Mexico, Albuquerque, New Mexico, United States of America; 2 John A. Burns School of Medicine, University of Hawaii at Manoa, Honolulu, Hawaii, United States of America; 3 Instituto Conmemorativo Gorgas, Panama City, Panama; 4 National University of Mongolia, Ulaanbaatar, Mongolia; 5 National Center for Emerging and Zoonotic Infectious Diseases, Centers for Disease Control and Prevention, Atlanta, Georgia, United States of America; George Washington University, UNITED STATES

## Introduction

A series of emerging pathogen outbreaks during the past 24 months (e.g., Ebola virus disease, Middle East respiratory syndrome, and Zika virus-associated microcephaly, and Guillain-Barre syndrome) have commanded the public’s attention and have exposed gaps in our preparedness to rapidly respond to these challenges. For example, the disease prevention and vector control response to the introduction and local spread of Zika virus infection in the United States is being blunted and hampered by congressional discord. Also, relying on legislation for emergency funds for each outbreak (rather than having a dedicated budget for preparedness and response to infectious disease outbreaks) is problematic. That said, previous zoonotic pathogen crises provide valuable insights into best practices, and herein, we detail the role of museum biorepositories in disease outbreak investigations. In addition to providing wide taxonomic sampling, museums and associated databases critically tie discoveries of new pathogens to permanent host records and samples and to a series of other informatics resources (e.g., GenBank and GIS applications) that facilitate future exploration, tracking, and mitigation of novel zoonotic pathogens. Because a fundamental requirement for the designation of a new pathogen is precise identification of the reservoir taxon [1], we advocate formal incorporation of museum biorepositories and integrated databases as critical infrastructure for pathogen discovery and pathobiology research.

## Case Study

**Fig 1:**
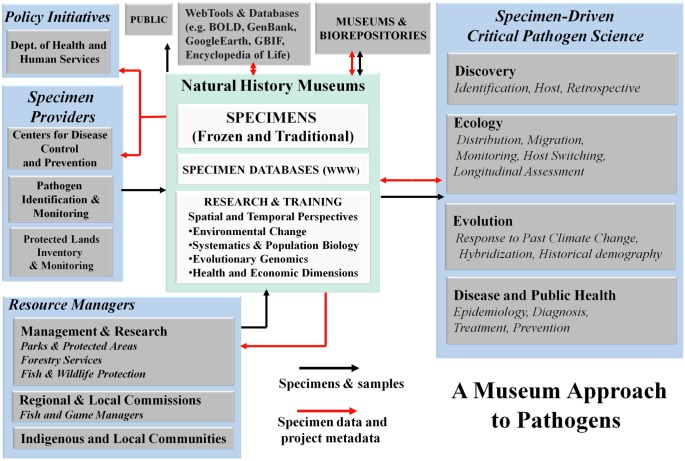
A museum-biorepository–based model for pathogen discovery and pathobiology research.

Approximately 40 years have passed since the identification of the striped field mouse (*Apodemus agrarius*) as the reservoir host of Haantan virus, the prototype virus of the genus *Hantavirus* in the family Bunyaviridae [2]. However, significant gains in our understanding of these pathogens did not occur until 1993, when an outbreak of a rapidly progressive, frequently fatal respiratory disease, now known as hantavirus pulmonary syndrome, was caused by Sin Nombre virus, a hantavirus harbored by the deer mouse (*Peromyscus maniculatus*) in the southwestern US [3]. That outbreak marked the beginning of integrated collaborations between public health agencies, virologists, ecologists, and museum scientists that completely reshaped our understanding of hantavirus systematics, evolution, and ecology. This interdisciplinary approach serves as a new model for pathogen discovery (Fig 1) and will be critical going forward as zoonotic pathogens and diseases emerge in the future [4]. Frozen tissues held in natural history museums stimulated discovery of many new hantaviruses in rodents (and, more recently, in shrews, moles, and bats) worldwide [5–6].

## Biorepositories and Databases

**Fig 2:**
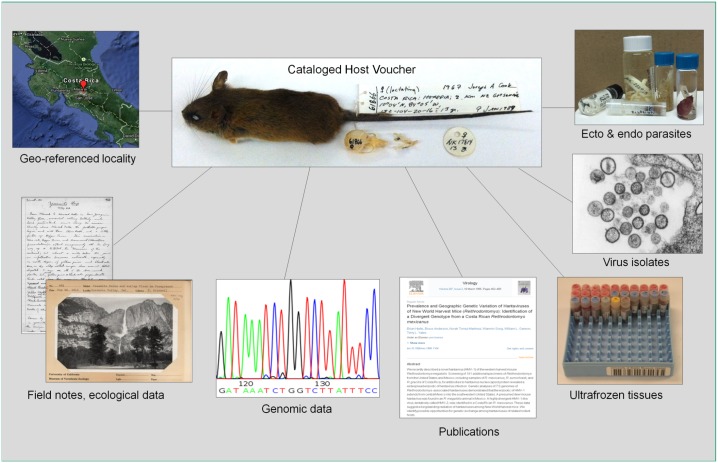
Pathogen symbiotype. Geo-referenced and time-stamped host specimen deposited in an accredited museum and linked through a single museum catalog number to ecological data, associated parasites, microbial pathogens, frozen tissues, genomic data, and publications derived from these materials.

Biomedical and pathobiology communities increasingly rely on archived human specimens to retroactively explore questions related to the etiology and pathogenesis of human diseases. Similarly, availability of frozen archives of wild vertebrates in museums permits rapid and efficient screening for diverse zoonotic pathogens and represents a major step forward in assessment, prevention, and mitigation of emerging diseases. Museum biorepositories have rigorous archival and database standards that ensure best practices are followed in pathogen discovery [7]. When new pathogens are described, permanent designation and deposition of host symbiotypes [8] provides a permanent link between samples and data (Fig 2). Macroparasites and microparasites present additional complexity due to their intimate association with particular host taxa. Host–parasite relationships critically require formal recognition to ensure not only that the original sample persists into the future but also that the identity of the pathogen reservoir will not be lost during the dynamic process of taxonomic revision.

Recent analyses have revised the taxonomy of many zoonotic pathogen reservoirs, work that was only possible because the original host vouchers were preserved and available in museums. Many other species that serve as pathogen reservoirs are in need of critical taxonomic revision. For viruses, identification of reservoir species is often problematic (e.g., Ebola virus). Therefore, in-depth knowledge of potential hosts, their taxonomic affinities and relationships, and geographic distributions is vital [9]. We recommend several standardized procedures for integrating museum biorepository infrastructure into pathogen research (Table 1).

**Table 1:** Recommendations.

1. **Symbiotype designation**: A single host specimen from which the novel pathogen was sequenced and/or isolated, and then described, should be formally designated. Taxonomy, museum catalog number (e.g., MSB:Mamm:89863), geo-referenced collection locality, date of collection, and institution of deposition should be included in the original publication.
2. **Symbiotype deposition**: Specimen, tissues, RNA and DNA extracts, and other ancillary material and data should be deposited and catalogued in an accredited natural history museum where all material will be permanently archived and available for future use by qualified investigators.
3. **Pathogen name**: Symbiotype catalog number should be included in the pathogen name (e.g., Camp Ripley virus [RPLV] MSB89863) to facilitate linkage.
4. **GenBank accessions**: Symbiotype identity should be confirmed with a DNA sequence (e.g., cytochrome *b* for mammals) deposited in GenBank. Both symbiotype and pathogen accession records should report the catalog number (e.g., MSB:Mamm:89863) in the “Definition” and “Specimen Voucher” data fields.
5. **Database**: The archiving institution should maintain a relational, web-accessible database (e.g., Arctosdb.org) linked to major biodiversity information servers (i.e., VertNet, GBIF, iDigBio) and directly to GenBank.
6. **Archiving institution**: Symbiotypes should be identified and managed as type specimens in the museum biorepository. Color-coded labels and notation in databases should identify the specimen as such. Traditional voucher material should be stored in a type case, and tissues should be held in a type rack in liquid nitrogen or -80°C freezers.
7. **Symbiotype/pathogen list**: List of symbiotypes and described pathogens held in a collection should be published or made available online.
8. **Specimens examined and serology results**: Should be included in publication or available as supplementary material.

Although the fundamental utility of host voucher specimens and frozen tissue collections is recognized and has been championed by a few disease ecologists [10], wide acceptance of the concept is still lacking. Science advances as hypotheses are tested, experiments are replicated, and accumulated knowledge is reinterpreted in light of new information, tools, and analyses. Future availability of samples that produced the original, primary data is critical should questions arise regarding their nature, provenance, or taxonomic identity [11]. Over time, a single archived specimen (and associated GenBank sequence) may integrate across dozens of projects and subsequent publications [12], but because most GenBank accessions are not linked to specimens, we are too often unable to replicate or confirm data. With more than 20% of GenBank data potentially misidentified [13], the gold standard for GenBank accessions is now based on the voucher specimen concept [14]. We further advocate that all zoonotic pathogen descriptions provide molecular identification (nucleic acid sequence) for both the host and pathogen so that their identities can readily be placed on the Tree of Life [15] and provide a basis for identifying sister species that may serve as potential hosts for related pathogens.

## Future Directions

Field collections of natural history specimens often arise through dynamic collaborations that are capable of producing a diverse array of preparations and associated data (e.g., ultra-frozen tissue, cell suspensions, feces, and endo- and ecto-parasites) with precise spatial and temporal stamps that facilitate myriad investigations. When properly archived and digitally captured, museum databases are capable of linking diverse kinds of “big data.” This biorepository nexus can be a powerful tool for research in pathogen discovery, environmental change, and host–reservoir dynamics. Spatially broad and temporally deep archives of ultra-frozen tissues represent unparalleled infrastructure for virologists, as demonstrated through the retrospective surveys for Sin Nombre hantavirus [16] and subsequent significant new hantavirus discoveries across four continents [5–6]. As tools for extracting vast amounts of information from both contemporary and ancient specimens improve [17], new insights into pathogen evolution and ecology will be enhanced [18]. We suggest that the benefits of incorporating this model into pathogen discovery and pathobiology research far outweigh any potential costs associated with its implementation (Box 1).

**Box 1. Advantages and Disadvantages of Museum Biorepositories and Integrated Databases**

**Advantages:**
- Maintains spatially broad, temporally deep and site-intensive archives of ultra-frozen vertebrate tissues
- Permanently links host specimens and tissues, microbial and host genetic sequences, associated publications, and other related data or materials
- Ensures that pathogen reservoir identity is not lost due to taxonomic revision
- Establishes best practices for loan agreements and specimen tracking
- Facilitates inclusion of museum catalog numbers in GenBank accessions prior to accepting manuscripts for publication

**Disadvantages:**
- Necessitates long-term institutional commitment to support personnel and physical infrastructure
- Requires periodic inventory of the number and condition of biospecimens
